# The adaptive immune and immune checkpoint landscape of neoadjuvant treated esophageal adenocarcinoma using digital pathology quantitation

**DOI:** 10.1186/s12885-020-06987-y

**Published:** 2020-06-01

**Authors:** Matthew P. Humphries, Stephanie G. Craig, Rafal Kacprzyk, Natalie C. Fisher, Victoria Bingham, Stephen McQuaid, Graeme I. Murray, Damian McManus, Richard C. Turkington, Jacqueline James, Manuel Salto-Tellez

**Affiliations:** 1 Precision Medicine Centre of Excellence, Patrick G Johnston Centre for Cancer Research, Queen’s University, Belfast, UK; 2 Cellular Pathology, Belfast Health and Social Care Trust, Belfast City Hospital, Lisburn Road, Belfast, UK; 3 Northern Ireland Biobank, Centre for Cancer Research and Cell Biology, Queen’s University, Belfast, UK; 4 Pathology, School of Medicine, Medical Sciences and Nutrition, University of Aberdeen, Aberdeen, Scotland; 5 Patrick G Johnston Centre for Cancer Research, Queen’s University, Belfast, UK

**Keywords:** Esophageal adenocarcinoma, Immune checkpoint, Multiplex IHC, Image analysis

## Abstract

**Background:**

Limited studies examine the immune landscape in Esophageal Adenocarcinoma (EAC). We aim to identify novel associations, which may inform immunotherapy treatment stratification.

**Methods:**

Three hundred twenty-nine EAC cases were available in Tissue Microarrays (TMA) format. A discovery cohort of 166 EAC cases were stained immunohistochemically for range of adaptive immune (CD3, CD4, CD8 and CD45RO) and immune checkpoint biomarkers (ICOS, IDO-1, PD-L1, PD-1). A validation cohort of 163 EAC cases was also accessed. A digital pathology analysis approach was used to quantify biomarker density.

**Results:**

CD3, CD4, CD8, CD45RO, ICOS and PD-1 were individually predictive of better overall survival (OS) (Log rank *p* = < 0.001; *p* = 0.014; *p* = 0.001; *p* = < 0.001; *p* = 0.008 and *p* = 0.026 respectively). Correlation and multivariate analysis identified high CD45RO/ICOS patients with significantly improved OS which was independently prognostic (HR = 0.445, (0.223–0.886), *p* = 0.021). Assessment of CD45RO and ICOS high cases in the validation cohort revealed an associated with improved OS (HR = 0.601 (0.363–0.996), *p* = 0.048). Multiplex IHC identified cellular co-expression of high CD45RO/ICOS. High CD45RO/ICOS patients have significantly improved OS.

**Conclusions:**

Multiplexing identifies true cellular co-expression. These data demonstrate that co-expression of immune biomarkers are associated with better outcome in EAC and may provide evidence for immunotherapy treatment stratification.

## Background

Esophageal cancer (EAC) incidence has increased several fold in the last 30 years and is responsible for over four hundred thousand deaths globally per annum (http://www.cancerresearchuk.org/health-professional/oesophageal-cancer-statistics), with adenocarcinoma now the dominant histology reported in the Western world [1]. As well as surgery, the current treatment regimens include cytotoxic chemotherapy and radiation and are associated with significant morbidity. Five year survival rates remain poor at 17% and for resectable disease this figure remains at approximately 45% despite improvements in surgery and neo-adjuvant therapy. The wide variability in patient outcome following resection is indicative of the need for robust biomarkers for prognostication, even following the improved survival rates seen with the introduction of neo-adjuvant chemotherapy [2, 3]. There is an urgent need for targeted therapies and deeper understanding of EAC.

Response to checkpoint inhibition has been shown to be more effective in tumours with a higher mutational load [4]. These tumour types are increasingly being considered as potential targets for immunotherapy. EAC is one example of a tumour type with a high mutational frequency with as many as three hundred thousand mutations per tumour [5].

The immune context of the tumour microenvironment (TME) comprise a range of regulatory proteins which, upon activation, can inhibit effective T-cell response leading to antitumor immunity. This regulation is a vital checkpoint in the inflammatory cascade, which many tumour types are able to evade. However, there have been recent success with checkpoint inhibition in solid tumours where increased expression of checkpoint proteins has been associated with a poor clinical outcome [6, 7]. This has been specifically demonstrated for EAC [8–10]. There is evidence that PD-L1 expression in EAC tumours is predictive of response in phase 1 trials, NCT01928394, NCT01943461 and NCT01772004 reported in a recent meta-analysis [11]. Nevertheless, increasing data suggest that expression of PD-L1 alone may not be adequate to be predict response due to the lack of response seen in many PD-L1 expressing tumours [12, 13]. This is further compounded by responses reported in PD-L1 negative cases [14]. Consequently, there has been a shift in focus to the immune contexture of the TME to identify cell phenotypes which may serve as prognostic biomarkers [15, 16]. These efforts including identification, quantification and spatial analysis of tumour-infiltrating lymphocytes and other immune and immune checkpoint biomarkers.

We report here on the immunohistochemical expression of several T cell linage markers and immune checkpoint biomarkers and their combinatorial influence on EAC outcome. We highlight a potential therapeutic advantage for a subset of patients and discuss the survival impact of immunological factors.

## Methods

### Ethical approval, patient material and tissue microarray creation

Following ethical approval, a discovery cohort of 166 EAC cases were made available across seven TMAs. Prior to surgical resection, patients received neo-adjuvant chemotherapy. Median follow up time was 48.8 months. Clinicopathological details are shown in Table 1 and have recently been described for the cohort [17, 18]. Of these 166 cases, 145 cases had sufficient tumour volume and biomarker data available for analysis with matched clinical information. Cases were diagnosed in Northern Ireland from 2004 to 2012 with ethical approval granted by the Northern Ireland Biobank (NIB15/0168) and ORECNI (13-NI-0149) [19]. A validation set of 163 resected gastro-oesophageal adenocarcinoma cases represented on five TMA was obtained from the Grampian biorepository, diagnosed within the Grampian National Health Service Scotland from 2004 to 2010, accessed through the Grampian Biorepository (OREC 18/LO/0161), which has been previously described [20].

**Table 1:** Clinicopathological data for discovery and validation cohorts

Characteristics	Discovery TMA *N* = 145 (%)	Validation TMA *N* = 163 (%)
**Age**		
< 60	42 (29%)	47 (29%)
= > 60	103 (71%)	115 (71%)
Unknown	0 (0%)	1 (< 1%)
**Sex**		
Male	115 (79%)	117 (72%)
Female	30 (21%)	46 (28%)
**Tumour site**		
Esophagus	8 (6%)	71 (44%)
Stomach	0 (0%)	92 (56%)
GOJ, Siewert 1	81 (56%)	0 (0%)
GOJ, Siewert 2	44 (30%)	0 (0%)
GOJ, Siewert 3	12 (8%)	0 (0%)
**T Stage**		
pT0/1	3 (2%)	24 (15%)
pT2	11 (8%)	55 (34%)
pT3	117 (81%)	81 (49%)
pT4	2 (1%)	3 (2%)
Unknown	12 (8%)	0 (0%)
**N Stage**		
N0	33 (23%)	72 (44%)
N1	85 (59%)	74 (45%)
N2/3	3 (2%)	17 (11%)
Unknown	24 (16%)	0 (0%)
**Lymph node**		
Positive	97 (67%)	91 (56%)
Negative	48 (33%)	72 (44%)
**Mandard TRG**		
1	1 (< 1%)	0 (0%)
2	8 (6%)	6 (4%)
3	28 (19%)	14 (9%)
4	69 (48%)	25 (15%)
5	33 (23%)	20 (12%)
Unknown	6 (4%)	67 (41%)
No Chemotherapy	0 (0%)	31 (19%)
**Margin Involvement**		
Positive	63 (43%)	39 (24%)
Negative	79 (55%)	124 (76%)
Unknown	3 (2%)	0 (0%)
**Neo-adjuvant chemotherapy**		
Yes	145 (100%)	85 (52%)
No	0 (0%)	66 (41%)
Missing	0 (0%)	12 (7%)

The construction of the TMAs was three 1-mm diameter cores were taken from the central tumour region from each representative resection tumour FFPE block in the discovery cohort and two 1-mm diameter cores were taken from the central tumour region for the validation cohort. Cores were only taken from cases containing tumour. Tumours with regression and regions containing scarring or inflammation only were not sampled. TMAs were created using a Beecher Manual Arrayer®.

### Immunohistochemistry

Immunohistochemical analysis was performed within the Precision Medicine Centre of Excellence. Slides were immunostained on a Ventana BenchMark or Leica Bond Rx fully automated immunostainers, with previously validated antibodies for CD3, CD4, CD8, CD45RO, ICOS, IDO-1, PD-L1 and PD-1 (Table S1). Briefly, antigen retrieval using cell conditioning solution 1 (CD3, CD4, PD-L1 and PD-1) or epitope retrieval solution 2 (CD8, CD45RO, ICOS, IDO-1) was performed. Optimised antibody concentrations were incubated according to the manufacturer instructions. Bound antibody was detected with Ultraview DAB/Optiview DAB Kits (Roche) or Bond Polymer Refine Detection Kit (Leica). Following initial microscopic quality control, all slides were digitised on a Leica Aperio AT2 scanner at × 40 magnification.

Multiplexing of CD45RO/ICOS/Cytokeratin (CK)/DAPI was conducted on a Leica Bond Rx fully automated immunostainer, with validated antibodies for CD45RO, ICOS and CK using the Opal Multiplex Immunostaining kit (PerkinElmer; Waltham, MA) according to the manufacturer’s instructions (Supplementary Table S1). Briefly, antigen retrieval was performed using epitope retrieval solution 2, validated CK was applied followed by the Opal 520 fluorophore at the working concentration. Unbound antibody was removed with heat-meditated retrieval. The process was repeated for two additional rounds for CD45RO and ICOS followed by secondary labelling with the respective fluorophores (Opal 570 and Opal 690, respectively). Nuclei were labelled with DAPI for 10 min. Immunofluorescent slides were digitised on a Vectra Polaris fluorescent scanner at × 40 magnification.

Optimised DAB biomarker expression was evaluated by and agreed upon with a senior consultant pathologist prior to the study. Assessment of each DAB biomarker was undertaken using open-source software QuPath [21]. Assessment of fluorescent Multiplex slides was conducted using the Vectra Polaris inForm software according to established workflows. Digital image analysis was carried out, blinded to clinical data, according to cell detection algorithms defined for each biomarker under the direction of senior consultant pathologists and/or a senior principal clinical scientist (MST, JJ and SMcQ) to determine the number of positive cells per mm^2^. Briefly, TMAs were de-arrayed, tissue detection was performed by identifying the tissue for cellular analysis. Samples containing less than 100 tumour cells were excluded from analysis. Rigorous quality control (QC) steps were taken to remove necrosis or keratin, tissue folds, entrapped normal structures and confirm data consistency across TMAs cores from the same tumour; this was confirmed by a second reviewer with frequent consultation, with case consensus decisions taken if TMA core data was highly discordant, following an established method, previously described [21–25].

This study was performed and reported in line with the REMARK criteria [26].

### Statistical analysis

Biomarkers were dichotomised using a cut off (Supplementary Table S2) derived by receiver operator curve (ROC) employing Euclidean distance methodology to determine optimum sensitivity and specificity value. Associations with Overall Survival (OS) (OS; from initial diagnosis to death) were analysed by Kaplan–Meier survival analysis, reporting log rank *p* values. Patients were censored at the last day they were known to be alive. Variables were entered in univariate and multivariate analysis included Mandard tumour regression grading, T stage, N Stage, nodal status and circumferential resection margin involvement defined as tumour cells at or within 1 mm of the margin, as is routinely used in analysis of EAC datasets. Hazard ratios (HR) and *P* values reported for univariate and multivariate analysis were calculated using Cox proportional hazards regression model. All statistics were carried out in IBM® SPSS (v25).

## Results

### Validated biomarker expression

**Fig. 1:**
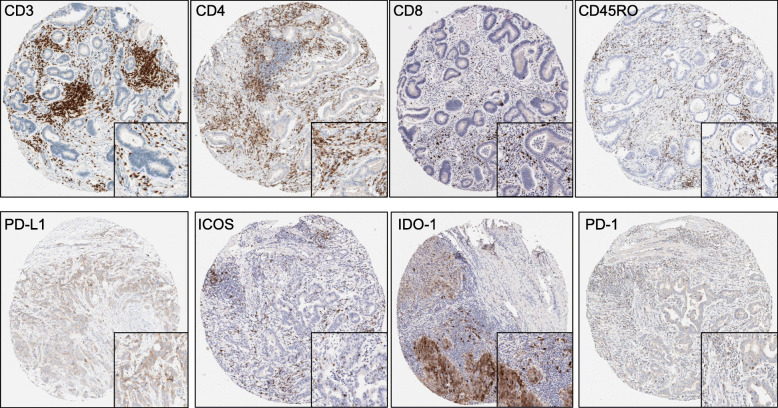
Optimised biomarkers staining for the immune and immune checkpoint proteins quantified. Each TMA core (10x) shows a case where DAB staining was clear and quantifiable. Each panel contains a magnified view of the staining at 40x

Cellular expression of CD3, CD4, CD8 and CD45RO was observed as strong membrane staining as expected, with characteristic, strong and fine, membrane staining seen for the PD-L1 clone SP263. ICOS, IDO-1, PD-1 displayed both nuclear and cytoplasmic staining with a weak cytoplasmic blush. Examples of optimised and validated staining on TMA cores are shown in Fig. 1.

### Immune checkpoint biomarkers impact overall survival

**Fig. 2:**
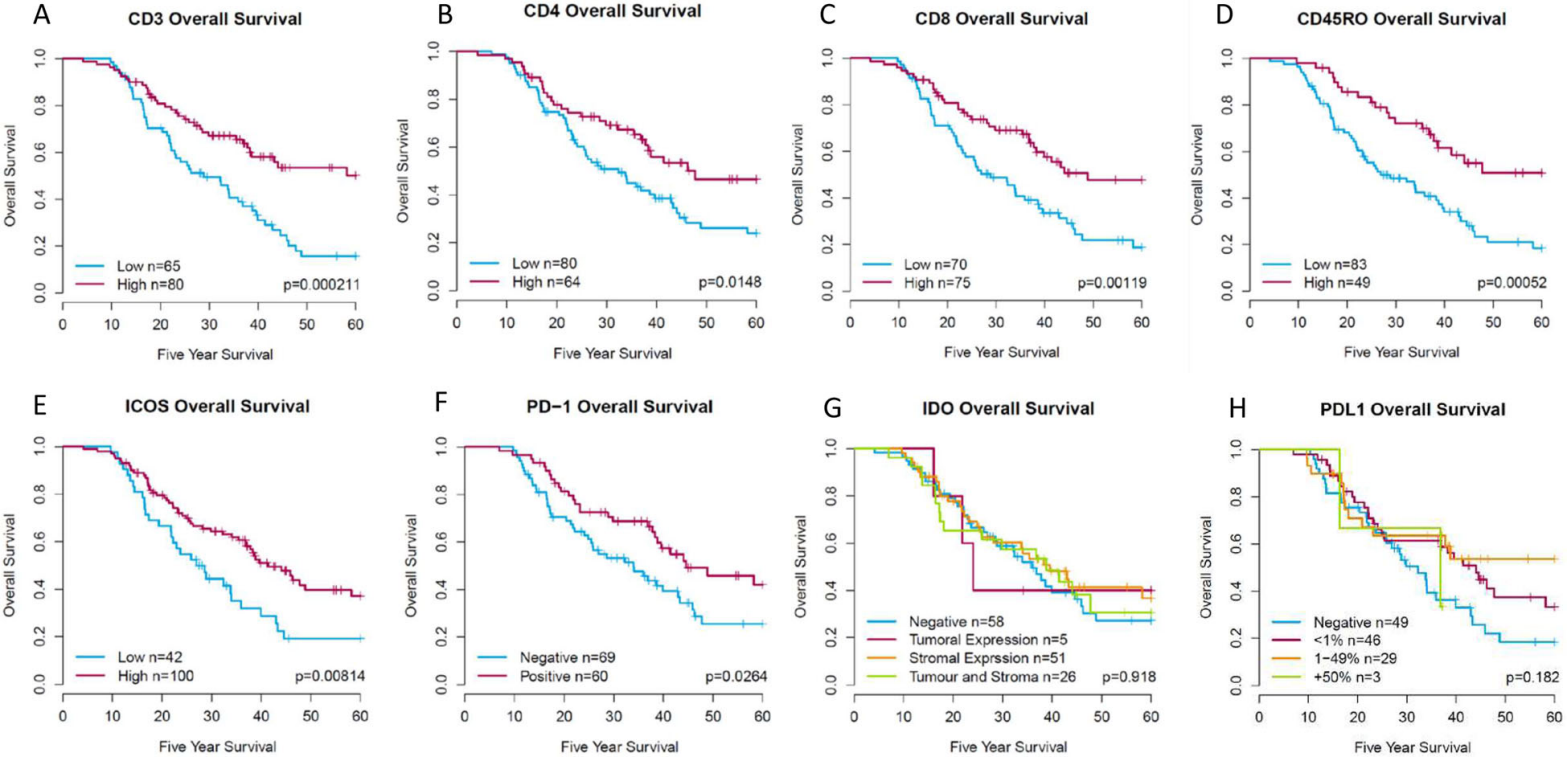
Adaptive immune and Immune checkpoint biomarker expression impact on five-year patient survival. Dichotomisation was based on the cut-off calculated using a ROC curve. Cases falling into the high group are represented by the red line with the blue line representing low expression for **a** CD3, **b** CD4, **c** CD8, **d** CD45RO, **e** ICOS and **f** PD-1. **g** IDO-1, was dichotomised based on expression of the biomarker in tumour, stroma or both, while **h** PD-L1, was grouped based on clinically established cut-off points (< 1%, 1–49 and > 50%). For IDO-1 and PD-L1 the dichotomisation is represented in the key for each biomarkers respectively. Log-rank *p* values are shown for each graph

Whole core scores were assessed for each single IHC biomarker and were generated using a robust digital pathology workflow, figure S1. Biomarkers in the discovery cohort were quantified by the density of positive cells per mm^2^ for CD3, CD4, CD8 and CD45RO. Kaplan Meier survival analysis showed that high CD3, CD4, CD8 and CD45RO were significantly associated with better OS (Fig. 2a, b, c and d, Log rank *p* = < 0.001; 0.001; 0.001 and < 0.001, respectively).

Following assessment of adaptive immune biomarkers, a series of immune checkpoint biomarkers (ICOS, IDO-1, PD-1 and PD-L1) were also assessed in the discovery cohort by density of positive cells per mm^2^, with the exception of IDO-1 and PD-L1. PD-L1 scoring was assessed by percentage tumour positivity using established clinically relevant thresholds for non-small cell lung cancer (NSCLC) (< 1%, 1–49% or > 50% tumour epithelial cell positivity). Due to the low number of cases positive for IDO-1 manual assessment in the tumour only, stroma only and whole core was the most optimal method using a (0 = 0%, 1 = 1–33%, 2 = 34–66%, 3 ≥ 66%) scoring system reported for IDO-1 assessment in breast cancer tissue [27]. Kaplan Meier survival analysis showed that high expression of ICOS and PD-1 but not IDO-1 and PD-L1 were associated with significantly better OS (Fig. 2e, f, g and h, Log rank *p* = 0.008; 0.026; 0.918 and 0.182, respectively). IDO-1 expression was observed in several cases to be exclusively expressed within intra-tumoural nests or exclusively to the stroma, as well as combinations of both. Therefore, tissue compartment expression assessment was undertaken for IDO-1. There was no impact on OS for IDO-1 irrespective of cellular location (Fig. 2g). Adaptive immune and immune checkpoint associations by univariate analysis are shown in Table 2.

**Table 2:** Univariate and Multivariate analysis of clinicopathological factors and immune and immune checkpoint biomarkers in EAC in the discovery and validation cohorts

Variables	Discovery		Validation	
	HR (95% CI)	*p*-value	HR (95% CI)	*p*-value
**Univariate Analysis**				
Mandard	1.36 (1.032–1.792)	**0.029**	1.833 (1.223–2.898)	**0.004**
T Stage	0.879 (0.7058–1.095)	0.25	1.764 (1.286–2.418)	**< 0.001**
N Stage	1.125 (0.7588–1.667)	0.558	1.473 (1.156–1.878)	**0.002**
Node Pos	1.03 (1.022–1.039)	**< 0.001**	2.180 (1.374–3.460)	**0.001**
CRM Involved	3.533 (2.238–5.576)	**< 0.001**	1.258 (1.093–1.447)	**0.001**
CD3	0.4521 (0.2929–0.6977)	**< 0.001**	–	–
CD4	0.6087 (0.3897–0.9509)	**0.029**	–	–
CD8	0.4932 (0.3188–0.7629)	**0.001**	–	–
CD45RO	0.4271 (0.2587–0.7052)	**< 0.001**	0.835 (0.546–1.278)	0.407
ICOS	0.5346 (0.3447–0.8291)	**0.005**	0.656 (0.424–1.015)	0.059
PD-1	0.540 (0.337–0.866)	**0.010**	–	–
IDO-1	0.95 (0.7935–1.137)	0.577	–	–
PD-L1	0.762 (0.570–10.19)	0.067	–	–
CD45RO/ICOS	0.394 (0.210–0.740)	**0.003**	0.601 (0.363–0.996)	**0.048**
**Multivariate Analysis**				
Mandard	1.321 (0.938–1.862)	0.111	2.078 (1.161–3.718)	**0.014**
T Stage	0.6846 (0.4968–0.943)	**0.020**	0.926 (0.397–2.160)	0.859
N Stage	1.264 (0.722–2.211)	0.412	1.759 (0.709–4.365)	0.223
Node Pos	1.0183 (1.006–1.0300)	**0.002**	0.482 (0.117–1.984)	0.312
CRM Involved	2.214 (1.2663–3.8711)	**0.002**	1.171 (0.842–1.628)	0.349
CD45RO/ICOS	0.445 (0.223–0.886)	**0.021**	0.810 (0.336–1.953)	0.639

**Fig. 3:**
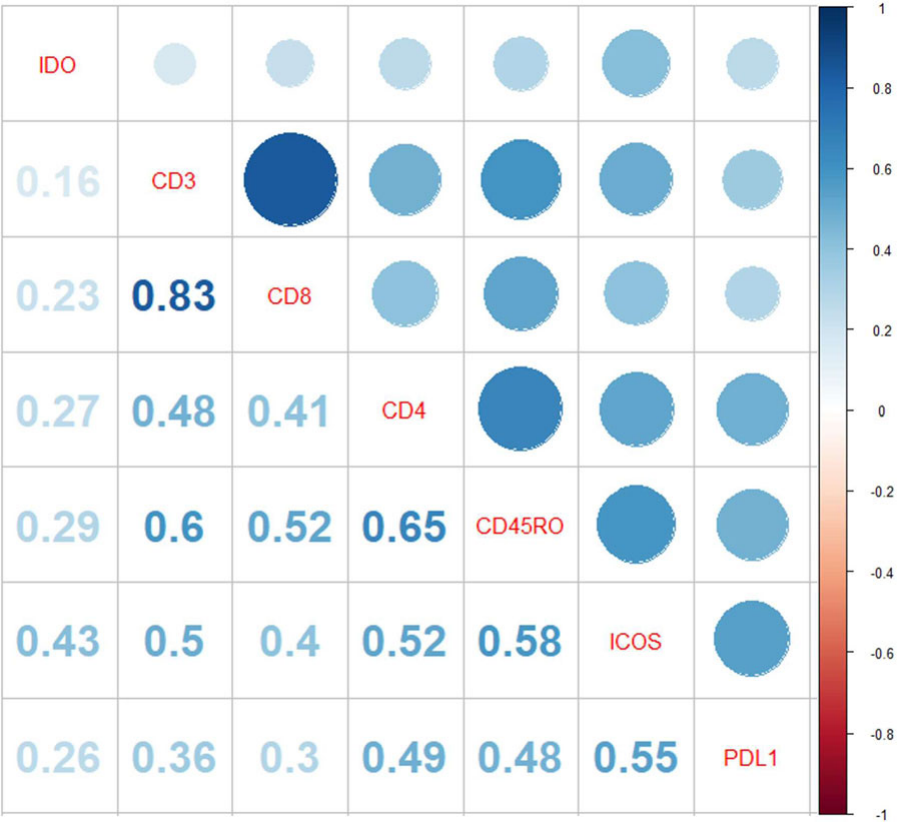
Correlation matrix displaying spearman rank. Positive correlation is represented in blue. The size of the circle corresponds to the magnitude of the correlation

To observe correlations between multiple biomarkers a correlation matrix was created displaying associations by spearman rank (Fig. 3). Positive correlations were represented in blue text with a circle corresponding to the magnitude of the correlation by size. Unsurprisingly, the highest correlation was CD3:CD8 (*r*^2^ = 0.83) while the lowest correlation was seen with CD3:IDO-1 (*r*^2^ = 0.16).

The impact of each immunological biomarker on OS was assessed in a multivariate model, which contained potential confounding clinicopathological factors. When evaluated in multivariate analysis CD45RO and ICOS co-expression remained independently prognostic (Table 2).

### Co-expression of immune biomarkers reveals immune hot and cold patient populations

**Fig. 4:**
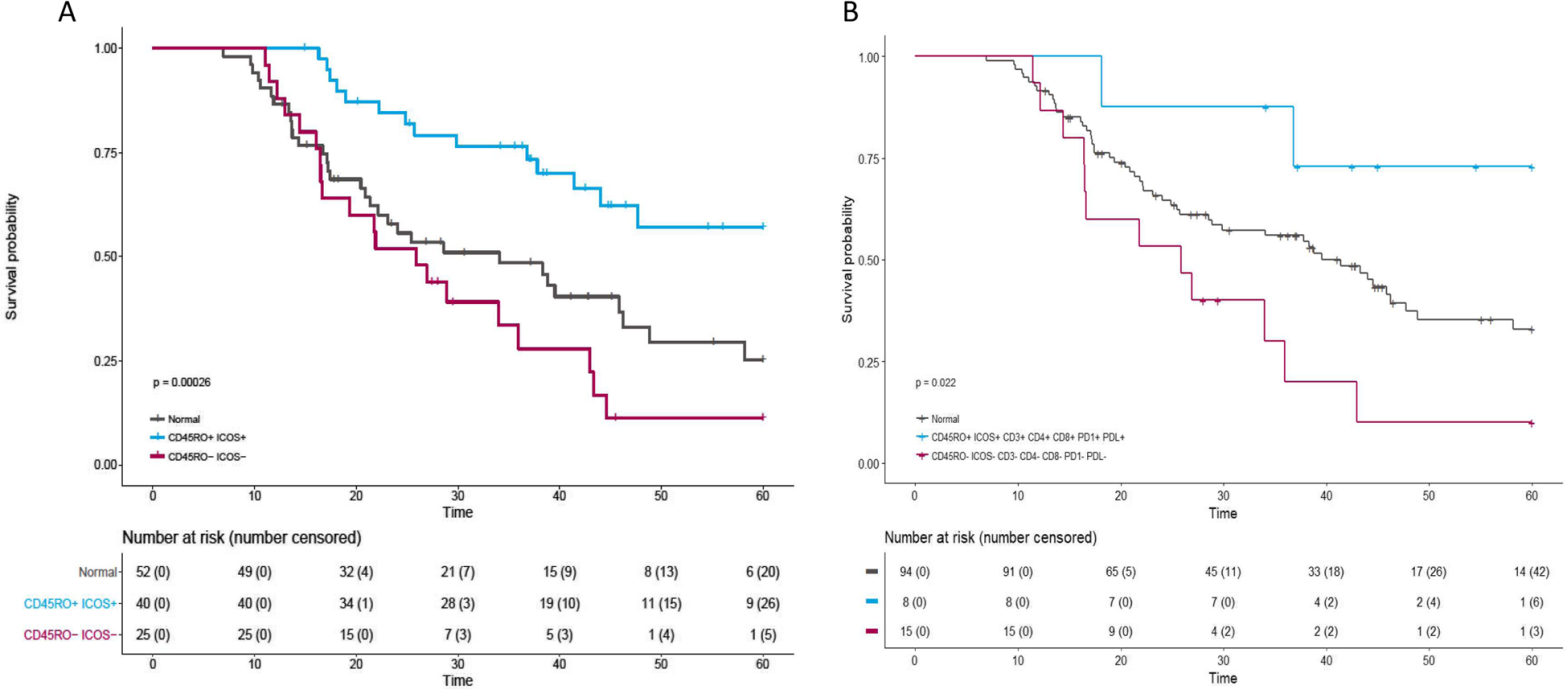
Five-year patient survival of ‘immune hot’ and high CD45RO/ICOS co-expressed cases in the discovery cohort. **a** Patients designated ‘immune hot’, expressing high level of CD45RO, ICOS, CD3, CD4, CD8, PD-1 and PD-L1 are in blue, contrasted with red where patented do not express these markers. **b** Patients expressing high CD45RO/ICOS, blue vs. low CD45RO/ICOS, red. Table below each graph displays number of patients in each group at specified time points, in brackets is the number of censored patients

Identification of a patient population, we described here as ‘immune hot’ (high CD3, CD4, CD8, CD45RO, ICOS, PD-1 and PD-L1), was observed. In contrast to a counter population of negatively expressing ‘immune cold’ cases, there was a striking variance in survival advantage for the ‘immune hot’ population (Fig. 4a). The ‘immune hot’ group had significantly better outcome over five-years compared to the ‘immune cold’ group, 74 and 11%, respectively (Log rank *p* = 0.022). However, due to the low number of cases for within these groups (*n* = 23), we must resist over-interpretation. As stated, CD45RO and ICOS remained independently prognostic in multivariate analysis. Therefore, examination of patient populations within this enriched phenotype of high CD45RO/ICOS was assessed against its contrasting population of low CD45RO/ICOS (Fig. 4b) (*N* = 65). Co-expression of high CD45RO and ICOS conferred a significant survival advantage over five-years, 65% (high/high) and 8% (low/low), respectively (Log rank *p* = < 0.001).

### High CD45RO/ICOS co-expression is positively prognostic in EAC

**Fig. 5:**
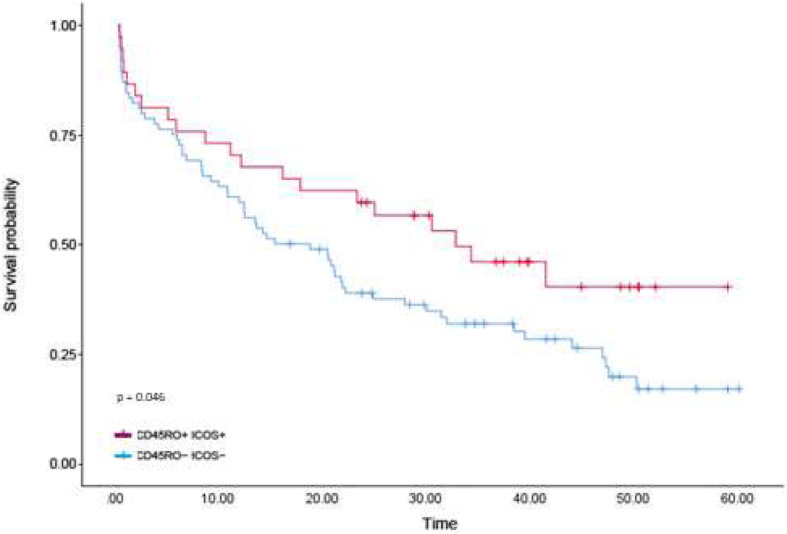
Five-year survival of patients expressing high CD45RO/ICOS vs low CD45RO/ICOS in the validation cohort. Cases falling into the high group are represented by the red line with the blue line representing low expression

Having identified the positive prognostication of CD45RO and ICOS co-expression in the discovery cohort, Table 2, which remained significant upon multivariate analysis (cox regression *p* = 0.021; HR, 0.445 (0.223–0.886), Table 2), and that demonstrated a high correlation, we examined the IHC expression in a validation cohort of a further 163 EAC [20]. The observed relationship in the discovery cohort was also seen in the validation cohort (log rank *p* = 0.045, Fig. 5; cox regression *p* = 0.048; HR, 0.601 (0.363–0.996), Table 2). This association however did not remain upon multivariate analysis in the validation set when adjusted for confounding factors (cox regression *p* = 0.639; HR, 0.810 (0.336–1.953), Table 2).

### Multiplexing reveals true cellular co-expression of immune biomarkers

**Fig. 6:**
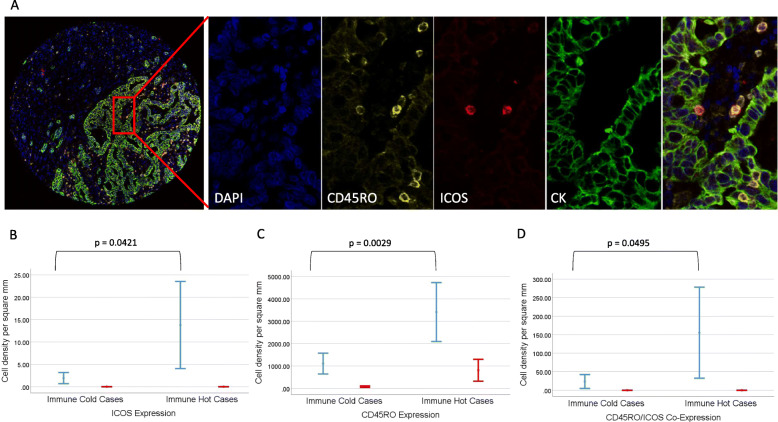
Multiplex co-expressing case displaying dual CD45RO/ICOS positive cell expression. **a** displays a TMA core multiplex image with an exploded view of a tumour and stromal region via individual fluorescence channels with dual labelled CD45RO/ICOS positive cells identified in the composite. **b** and **c** show graphically the assessment data for both ICOS and CD45RO, respectively, in both the tumour (blue) and stroma (red) for both ‘immune hot’ (*n* = 4) and ‘immune cold’ (*n* = 3) groups. **d** presents the cellular co-expression of CD45RO/ICOS positive cells within tumour (blue) and stroma (red) for both ‘immune hot’ (*n* = 4) and ‘immune cold’ (*n* = 3) groups. *P* values are two tailed t-tests assessing stromal biomarker expression across the two groups

In order to identify whether a population of truly high CD45RO/ICOS co-expressing cells is present in these patients, as opposed to a simultaneous increase in individually positive cells, a CD45RO/ICOS/CK/DAPI multiplex was conducted. Cases were identified from the prior determined ‘immune hot’ and ‘immune cold’ patient populations. Blinded to clinical data, whole core analysis was conducted digitally. Cellular co-expression was considered true when an individual cell was positive for both CD45RO and ICOS biomarkers (Fig. 6a). Tissue compartmental analysis demonstrated a statistically significant difference observed between the ‘immune hot’ group compared with the ‘immune cold’ for both stromal ICOS and stromal CD45RO, Fig. 6b and c respectively, paralleling the whole assessment in the chromogenic DAB IHC data, Fig. 2. We observed a significant difference in the cellular co-expression of positive CD45RO/ICOS cells within stroma in the ‘immune hot’ group compared with the ‘immune cold’ group (*p* = 0.049), Fig. 6d. There was no co-expression of CD45RO/ICOS positive cells within tumour nests, Fig. 6d.

## Discussion

Here, we described the immunophenotypic landscape of EAC and describe the existence of an ‘immune hot’ subgroup of patients that confers a significant survival advantage. Further delineation of only high CD45RO/ICOS cases was then investigated, informed by multivariate and correlative analysis, improving patient inclusion, stratification and OS advantage. We also describe, by multiplex immunofluorescence, co-expression of CD45RO/ICOS positive cells in these cases, observing a significant difference across the contrasting immune groups. Interrogation of dual labelled cell expression within the tumour and stroma revealed that stromal co-expression was significantly increased in the ‘immune hot’ group.

Efforts towards a better understanding the tumour-immune milieu of EAC are gradually increasing. We believe our collaborative study, inclusive of a validation EAC cohort, is the largest of its kind reported to date. Making these data one of very few comprehensive publications delineating immune biomarkers in EAC.

In some tumour types, NSCLC for example, there is extensive data supporting the assessment of PD-L1 protein expression as a good predictor of patient response to immunotherapy. The evidence is scant for such application in EAC, owing mainly to the wide-ranging expression patterns seen in EAC. Recent results of Keynote 62 study reported that pembrolizumab to first line chemotherapy did not show any benefit [13]. Though membranous epithelial expression has been reported in EAC, which was also observed in our study, PD-L1 expression has been predominantly observed in the surrounding tumour-stroma rather than the membranes of epithelial cells, as reported in KEYNOTE-012 and observationally by others [28–30]. This is in addition to the subjective and challenging pathological assessment of PD-L1, which may complicate the adoption of the diagnostic test [24]. Recently, negative prognostication of PD-L2 was reported for esophageal cancer. Though this is was mainly in squamous cell carcinoma, the authors found no significant correlation between PD-L2 and PD-L1 expression. However, these data may indicate evaluation of both PD-L2 and PD-L1 expression may be clinically important [31].

Attempts to assess densities of T cells in EAC have been made. The expression of high CD3, CD4, CD8 and FOXp3 cells in EAC was reported in 128 cases, where all markers achieved significance in univariate analysis, closely mirroring our own data. However, only high CD8 cells alone reached positive prognostic significance in multivariate analysis [32]. A similarly powered study demonstrated significance on OS by all markers (CD3, CD8 and FoxP3). Though CD8 positive T cell expression has been shown to be positively prognostic in EAC [33], Thompson et al*,* recently demonstrated that high CD8 infiltration was correlated with impaired progression free survival and OS [28]. These data directly contrast with our own, where we demonstrate, as with several aforementioned studies that increased CD8 densities correlated with improved OS. We believe disparities are likely due our increased cohort numbers, robust digital pathology quantification methodology and a statistically derived cut-offs in comparison to a lower sample numbers and alternatively defined dichotomisations (median and quartile thresholds).

We acknowledge weaknesses in our study such as the limited number of cases interrogated by multiplex, restricting our assessment of the impact on survival by these CD45RO/ICOS positive dual labelled cells. Nevertheless, in these few exemplar cases we demonstrate a statistically significant difference in expression of these dual labelled cells across ‘immune hot’ and ‘immune cold’ patient groups, which have individually and together shown survival impact by single chromogenic DAB IHC. The limited number of TRG 1 and 2 cases in our cohort has the potential to confound our analysis. To examine this, we undertook an analysis inclusive of only TRG3 and higher. No difference was observed and CD45RO/ICOS positive cases remained significantly positive prognostic by multivariate analysis (Supplementary Table S3). We acknowledge the increased number of gastric case in the validation cohort, which are potentially more inflammatory due to being potentially MSI high or EBV positive. MSI/EBV status is unexplored in this cohort. We also accept the retrospective, post-treatment nature of the cohort and recognise that preoperative treatment may likely to induce a permanent or transient change in the immune microenvironment of EAC [34]. We ideally would wish to compare and contrast the immune profiles of a surgery only cohort, without chemo/chemoradiation treatment, in comparison with our data here. With few patients going straight to surgery without some form of treatment, we are unable to obtain samples from a suitable historical cohort of sufficient size to explore this question. However, this cohort is representative of current clinical practice.

It is rational to assume that PD-L1 inhibitors may have applicability in EAC. However, studies have demonstrated contrasting prognostication of PD-L1 expression in EAC [8–10, 35]. Interestingly, as with PD-L1, we observe that ICOS, a member of the B7-family of proteins, as is PD-L1, was expressed predominantly in the stroma, conferring a positive prognostic survival advantage. Our data add support to the hypothesis that some of the more novel B7-family members could be regulating CD4 T-cell differentiation toward specific effector cell phenotypes. Indeed, ICOS/CD4 correlation has been reported in colorectal cancer, where expression is associated with improved survival and may be a clinical biomarker for good prognosis [36]. Here, we show the association of CD45RO/ICOS as being greater than that of ICOS/CD4 which maybe highlighting a previously unreported population of ICOS positive memory cells, which encompass CD4. The prognostication of high ICOS/CD4 cells may be being enhanced in our study, by evaluation of all CD45RO positive cells, which include; all memory T cells, B cell subsets, activated monocytes/macrophages and granulocytes, not solely a subpopulation of CD4 positive cells. Our data emphasise the scrutiny required when analysing experimental and trail data to consider 1) The specific cell phenotype of interest. 2) The expression of the target. 3) The expression of associated proteins and ligands. 4) The specificity of the antibody used. Our data may yet be corroborated in clinical trial data with the ICOS agonist GSK3359609 (GlaxoSmithKline, London, UK), currently evaluated in a phase I trial INDUCE-1, NCT02723955. As well as JTX-2011, (Jounce Therapeutics, Cambridge, USA) in phase I/II ICONIC trial, NCT02904226. Future work aims to assess CD45RO/ICOS positive dual labelled cells by multiplexing in a large cohort of cases to assess impact on survival.

## Conclusion

We describe the existence of both ‘immune hot’ and ‘immune cold’ patient populations in EAC. Several immune biomarkers examined were shown to be positively prognostic for OS. Upon correlative and multivariate analysis, we identified a high CD45RO/ICOS subgroup of patients, with co-expression of these biomarkers, stratifying patients in to contrasting survival groups. Multiplexing revealed dual labelled CD45RO/ICOS positive cells to be expressed in the stromal compartment of EAC tissue. These dual labelled cells were significantly differentially expressed across the ‘immune hot’ and ‘immune cold’ patient populations. In summary, we have identified a subpopulation of high CD45RO/ICOS EAC patients with significant positive prognostication. Moreover, these data provide evidence for the assessment of CD45RO/ICOS to be employed in the stratification of patients for clinical trials investigating the response to immunotherapy.

## Supplementary information

**Additional file 1: Table S1.** Antibody details.

**Additional file 2: Table S2.** R.O.C Cut Offs.

**Additional file 3: Table S3.** Discovery Cohort Multivariate Analysis Excluding TRG 1 and 2 cases.

**Additional file 4: Figure S1.** Robust digital pathology workflow. Flow diagram demonstrates conceptually the process of biomarker quantification, strict quality control and data validation steps.

## Data Availability

All data requests should be submitted to the corresponding author for consideration. Access to anonymised data may be granted following review.

## Abbreviations

EAC: Esophageal Adenocarcinoma
TMA: Tissue Microarrays
OS: Overall Survival
TME: Tumour Microenvironment
QC: Quality Control
ROC: Receiver Operator Curve
NSCLC: Non-Small Cell Lung Cancer

## References

[1] Brown LM, Devesa SS, Chow WH (2008). Incidence of adenocarcinoma of the esophagus among white Americans by sex, stage, and age. J Natl Cancer Inst.

[2] Cunningham D, Allum WH, Stenning SP, Thompson JN, Van DV NM, Scarffe JH, Lofts FJ, Falk SJ, Iveson TJ, Smith DB, Langley RE, Verma M, Weeden S, Chua YJ (2006). Perioperative chemotherapy versus surgery alone for Resectable Gastroesophageal Cancer. N Engl J Med.

[3] van Hagen P, Hulshof MCCM, van Lanschot JJB, Steyerberg EW, Henegouwen MI, Wijnhoven BPL, Richel DJ, Nieuwenhuijzen GAP, Hospers GAP, Bonenkamp JJ, Cuesta MA, Blaisse RJB, Busch ORC, ten Kate FJW, Creemers G-J, Punt CJA, Plukker JTM, Verheul HMW, Bilgen EJS, van Dekken H, van der Sangen, Rozema T, Biermann K, Beukema JC, Piet AHM, van Rij CM, Reinders JG, Tilanus HW, van der Gaast (2012). Preoperative Chemoradiotherapy for Esophageal or Junctional Cancer. N Engl J Med.

[4] Yarchoan M, Johnson BA, Lutz ER, Laheru DA, Jaffee EM (2017). Targeting neoantigens to augment antitumour immunity. Nat Rev Cancer.

[5] Lawrence MS, Stojanov P, Polak P, Kryukov GV, Cibulskis K, Sivachenko A, Carter SL, Stewart C, Mermel CH, Roberts SA, Kiezun A, Hammerman PS, McKenna A, Drier Y, Zou L, Ramos AH, Pugh TJ, Stransky N, Helman E, Kim J, Sougnez C, Ambrogio L, Nickerson E, Shefler E, Cortes ML, Auclair D, Saksena G, Voet D, Noble M, DiCara D, Lin P, Lichtenstein L, Heiman DI, Fennell T, Imielinski M, Hernandez B, Hodis E, Baca S, Dulak AM, Lohr J, Landau DA, Wu CJ, Melendez-Zajgla J, Hidalgo-Miranda A, Koren A, McCarroll SA, Mora J, Crompton B, Onofrio R, Parkin M, Winckler W, Ardlie K, Gabriel SB, CWM R, Biegel JA, Stegmaier K, Bass AJ, Garraway LA, Meyerson M, Golub TR, Gordenin DA, Sunyaev S, Lander ES, Getz G (2013). Mutational heterogeneity in cancer and the search for new cancer-associated genes. Nature.

[6] Krambeck AE, Thompson RH, Dong H, Lohse CM, Park ES, Kuntz SM, Leibovich BC, Blute ML, Cheville JC, Kwon ED (2006). B7-H4 expression in renal cell carcinoma and tumor vasculature: associations with cancer progression and survival. Proc Natl Acad Sci U S A.

[7] Sun JM, Zhou W, Choi YL, Choi SJ, Kim SE, Wang Z, Dolled-Filhart M, Emancipator K, Wu D, Weiner R, Frisman D, Kim HK, Choi YS, Shim YM, Kim J (2016). Prognostic significance of PD-L1 in patients with non-small cell lung Cancer: a large cohort study of surgically resected cases. J Thorac Oncol.

[8] Ohigashi Y, Sho M, Yamada Y, Tsurui Y, Hamada K, Ikeda N, Mizuno T, Yoriki R, Kashizuka H, Yane K, Tsushima F, Otsuki N, Yagita H, Azuma M, Nakajima Y (2005). Clinical significance of programmed death-1 ligand-1 and programmed death-1 ligand-2 expression in human esophageal cancer. Clin Cancer Res.

[9] Hatogai K, Kitano S, Fujii S, Kojima T, Daiko H, Nomura S, Yoshino T, Ohtsu A, Takiguchi Y, Doi T, Ochiai A (2016). Comprehensive immunohistochemical analysis of tumor microenvironment immune status in esophageal squamous cell carcinoma. Oncotarget.

[10] Chen L, Deng H, Lu M, Xu B, Wang Q, Jiang J, Wu C (2014). B7-H1 expression associates with tumor invasion and predicts patient's survival in human esophageal cancer. Int J Clin Exp Pathol.

[11] Khunger M, Hernandez AV, Pasupuleti V, Rakshit S, Pennell NA, Stevenson J, Mukhopadhyay S, Schalper K, Velcheti V (2017). Programmed cell death 1 (PD-1) ligand (PD-L1) expression in solid tumors as a predictive biomarker of benefit from PD-1/PD-L1 Axis inhibitors: a systematic review and meta-analysis. JCO Precision Oncology.

[12] Koemans WJ, Chalabi M, van Sandick JW, van Dieren JM, Kodach LL (2018). Beyond the PD-L1 horizon: in search for a good biomarker to predict success of immunotherapy in gastric and esophageal adenocarcinoma. Cancer Lett.

[13] Tabernero J, Van Cutsem E, Bang Y, Fuchs CS, Wyrwicz L, Lee KW, Kudaba I, Garrido M, Chung HC, Castro Salguero HR, Mansoor W, Braghiroli MIFM, Goekkurt E, Chao J, Wainberg ZA, Kher U, Shah S, Kang SP, Shitara K (2019). Pembrolizumab with or without chemotherapy versus chemotherapy for advanced gastric or gastroesophageal junction (G/GEJ) adenocarcinoma: the phase III KEYNOTE-062 study. JCO.

[14] Gibney GT, Weiner LM, Atkins MB (2016). Predictive biomarkers for checkpoint inhibitor-based immunotherapy. Lancet Oncol.

[15] Chen YP, Zhang Y, Lv JW, Li YQ, Wang YQ, He QM, Yang XJ, Sun Y, Mao YP, Yun JP, Liu N, Ma J (2017). Genomic analysis of tumor microenvironment immune types across 14 solid Cancer types: immunotherapeutic implications. Theranostics.

[16] Iglesia MD, Parker JS, Hoadley KA, Serody JS, Perou CM, Vincent BG. Genomic Analysis of Immune Cell Infiltrates Across 11 Tumor Types. J Natl Cancer Inst. 2016;108(11). 10.1093/jnci/djw144 Print 2016 Nov. 10.1093/jnci/djw144 PMC5241901 27335052

[17] Blayney JK, Cairns L, Li G, McCabe N, Stevenson L, Peters CJ, Reid NB, Spence VJ, Chisambo C, McManus D, James J, McQuaid S, Craig S, Arthur K, McArt D, Ong CJ, Lao-Sirieix P, Hamilton P, Salto-Tellez M, Eatock M, Coleman HG, Fitzgerald RC, Kennedy RD, Turkington RC, Oesophageal Cancer Clinical and Molecular Stratification (OCCAMS) Study Group (2018). Glucose transporter 1 expression as a marker of prognosis in oesophageal adenocarcinoma. Oncotarget.

[18] McMenamin UC, Trainor J, Coleman HG, McManus DT, McQuaid S, Bingham V, James J, Salto-Tellez M, Johnston BT, Turkington RC (2018). Sex hormone receptor expression and survival in esophageal adenocarcinoma: a prospective cohort study. Oncotarget.

[19] Lewis C, McQuaid S, Clark P, McGuigan T, Murray P, Greene C, Coulter B, Mills K, James J. The Northern Ireland biobank: a Cancer focused repository of science. Open J Bioresources. 2018;5:9. 10.5334/ojb.47.

[20] Hope NR, Murray GI (2011). The expression profile of RNA-binding proteins in primary and metastatic colorectal cancer: relationship of heterogeneous nuclear ribonucleoproteins with prognosis. Hum Pathol.

[21] Bankhead P, Loughrey MB, Fernandez JA, Dombrowski Y, McArt DG, Dunne PD, McQuaid S, Gray RT, Murray LJ, Coleman HG (2017). QuPath: open source software for digital pathology image analysis. Sci Rep.

[22] Bankhead P, Fernandez JA, McArt DG, Boyle DP, Li G, Loughrey MB, Irwin GW, Harkin DP, James JA, McQuaid S, Salto-Tellez M, Hamilton PW (2018). Integrated tumor identification and automated scoring minimizes pathologist involvement and provides new insights to key biomarkers in breast cancer. Lab Investig.

[23] Loughrey MB, Bankhead P, Coleman HG, Hagan RS, Craig S, McCorry AMB, Gray RT, McQuaid S, Dunne PD, Hamilton PW (2018). Validation of the systematic scoring of immunohistochemically-stained tumour tissue microarrays using QuPath digital image analysis. Histopathology.

[24] Humphries MP, McQuaid S, Craig S, Bingham V, Maxwell P, Maurya M, McLean F, Sampson J, Higgins P, Greene C, James J, Salto-Tellez M. Critical appraisal of PD-L1 reflex diagnostic testing: current standards and future opportunities. J Thorac Oncol. 2019;14(1):45–53. 10.1016/j.jtho.2018.09.025. 10.1016/j.jtho.2018.09.025 PMC6328626 30296485

[25] Humphries MP, Hynes S, Bingham V, Cougot D, James J, Patel-Socha F, Parkes EE, Blayney JK, O'Rorke MA, Irwin GW, McArt DG, Kennedy RD, Mullan PB, McQuaid S, Salto-Tellez M, Buckley NE (2018). Automated Tumour Recognition and Digital Pathology Scoring Unravels New Role for PD-L1 in Predicting Good Outcome in ER−/HER2+ Breast Cancer. J Oncol.

[26] McShane LM, Altman DG, Sauerbrei W, Taube SE, Gion M, Clark GM, Statistics Subcommittee of the NCI-EORTC Working Group on, Cancer Diagnostics (2005). REporting recommendations for tumour MARKer prognostic studies (REMARK). Br J Cancer.

[27] Soliman H, Rawal B, Fulp J, Lee J, Lopez A, Bui MM, Khalil F, Antonia S, Yfantis HG, Lee DH, Dorsey TH, Ambs S (2013). Analysis of indoleamine 2-3 dioxygenase (IDO1) expression in breast cancer tissue by immunohistochemistry. Cancer Immunol Immunother.

[28] Thompson ED, Zahurak M, Murphy A, Cornish T, Cuka N, Abdelfatah E, Yang S, Duncan M, Ahuja N, Taube JM, Anders RA, Kelly RJ (2017). Patterns of PD-L1 expression and CD8 T cell infiltration in gastric adenocarcinomas and associated immune stroma. Gut.

[29] Muro K, Chung HC, Shankaran V, Geva R, Catenacci D, Gupta S, Eder JP, Golan T, Le DT, Burtness B, McRee AJ, Lin CC, Pathiraja K, Lunceford J, Emancipator K, Juco J, Koshiji M, Bang YJ (2016). Pembrolizumab for patients with PD-L1-positive advanced gastric cancer (KEYNOTE-012): a multicentre, open-label, phase 1b trial. Lancet Oncol.

[30] Kawazoe A, Kuwata T, Kuboki Y, Shitara K, Nagatsuma AK, Aizawa M, Yoshino T, Doi T, Ohtsu A, Ochiai A (2017). Clinicopathological features of programmed death ligand 1 expression with tumor-infiltrating lymphocyte, mismatch repair, and Epstein-Barr virus status in a large cohort of gastric cancer patients. Gastric Cancer.

[31] Okadome K, Baba Y, Nomoto D, Yagi T, Kalikawe R, Harada K, Hiyoshi Y, Nagai Y, Ishimoto T, Iwatsuki M, Iwagami S, Miyamoto Y, Yoshida N, Watanabe M, Komohara Y, Shono T, Sasaki Y, Baba H (2020). Prognostic and clinical impact of PD-L2 and PD-L1 expression in a cohort of 437 oesophageal cancers. Br J Cancer.

[32] Noble F, Mellows T, McCormick Matthews LH, Bateman AC, Harris S, Underwood TJ, Byrne JP, Bailey IS, Sharland DM, Kelly JJ, Primrose JN, Sahota SS, Bateman AR, Thomas GJ, Ottensmeier CH (2016). Tumour infiltrating lymphocytes correlate with improved survival in patients with oesophageal adenocarcinoma. Cancer Immunol Immunother.

[33] Schumacher K, Haensch W, Roefzaad C, Schlag PM (2001). Prognostic significance of activated CD8(+) T cell infiltrations within esophageal carcinomas. Cancer Res.

[34] Kelly RJ, Zaidi AH, Smith MA, Omstead AN, Kosovec JE, Matsui D, Martin SA, DiCarlo C, Werts ED, Silverman JF, Wang DH, Jobe BA (2018). The dynamic and transient immune microenvironment in locally advanced esophageal adenocarcinoma post Chemoradiation. Ann Surg.

[35] Chen K, Cheng G, Zhang F, Zhang N, Li D, Jin J, Wu J, Ying L, Mao W, Su D (2016). Prognostic significance of programmed death-1 and programmed death-ligand 1 expression in patients with esophageal squamous cell carcinoma. Oncotarget.

[36] Zhang Y, Luo Y, Qin S, Mu Y, Qi Y, Yu M, Zhong M (2016). The clinical impact of ICOS signal in colorectal cancer patients. Oncoimmunology.

