# Supplementary material for: The adaptive immune and immune checkpoint landscape of neoadjuvant treated esophageal adenocarcinoma using digital pathology quantitation
Source: BMC Cancer. 2020 Jun 1;20:500. doi: 10.1186/s12885-020-06987-y (PMC7268770; doi:10.1186/s12885-020-06987-y)
Supplement: Supplementary file 1 — Additional file 1: Table S1. Antibody details. [file 12885_2020_6987_MOESM1_ESM.docx]

Supplemental Table S1 – Antibody details.

| **DAB Immunohistochemistry** | | | | | | | | |
| --- | --- | --- | --- | --- | --- | --- | --- | --- |
| Biomarker | Clone | Source | Platform | Antigen Retrieval | Dilution | Incubation | Detection chemistry | Control tissue |
| CD3 | 3GV6 | Ventana | Benchmark XT | CC1 for 32 minutes | Neat | 16 minutes at 37^O^C | Optiview DAB | Tonsil |
| CD4 | SP35 | Ventana | Benchmark XT | CC1 for 60 minutes | Neat | 16 minutes at 37^O^C | Ultraview DAB | Tonsil |
| CD45RO | UCHL1 | Leica | Lecia Bond RX | ER1 20MINS | 1:2000 | 15 minutes at room temperature | Bond Polymer Refine Detection and Enhancer | Tonsil |
| CD8 | CD/144B | Dako | Lecia Bond RX | ER2 for 20 minutes | 1:50 | 15 minutes at room temperature | Bond Polymer Refine Detection and Enhancer | Tonsil |
| ICOS | D1K2T | Cell Signalling | Lecia Bond RX | ER2 for 20 minutes | 1:400 | 15 minutes at room temperature | Bond Polymer Refine Detection and Enhancer | Tonsil |
| IDO-1 | D5J4E | Cell Signalling | Lecia Bond RX | ER2 for 20 minutes | 1:400 | 15 minutes at room temperature | Bond Polymer Refine Detection and Enhancer | Tonsil |
| PD-L1 | SP263 | Ventana | Benchmark XT | CC1 for 64 minutes | Neat | 20 minutes at room temperature | Optiview DAB | Tonsil |
| PD-1 | NAT105 | Ventana | Benchmark XT | CC1 for 30 minutes | Neat | 16 minutes at room temperature | Ultraview DAB | Tonsil |

| **Multiplex Immunofluorescence** | | | | | | | | | |
| --- | --- | --- | --- | --- | --- | --- | --- | --- | --- |
| Step | Biomarker | Clone | Source | Platform | Antigen Retrieval | Dilution | Incubation | Detection chemistry | Control tissue |
| 1 | CK | AE1/AE3 | DAKO | Lecia Bond RX | ER2 20min | 1:100 | 30 minutes at room temperature | Opal 520 @ 1:150 for 30mins | Tonsil |
|  |  |  |  |  |  |  |  |  |  |
| 2 | CD45RO | UCHL1 | Leica | Lecia Bond RX | ER1 20min | 1:2000 | 30 minutes at room temperature | Opal 570 @ 1:150 for 30mins | Tonsil |
|  |  |  |  |  |  |  |  |  |  |
| 3 | ICOS | D1K2T | Cell Signalling | Lecia Bond RX | ER2 20min | 1:400 | 30 minutes at room temperature | Opal 690 @1:150 for 30mins | Tonsil |
| 4 | DAPI | n/a | Perkin Elmer | Lecia Bond RX | n/a | 1:500 | 30 minutes at room temperature | n/a | Tonsil |
