# Supplementary material for: The adaptive immune and immune checkpoint landscape of neoadjuvant treated esophageal adenocarcinoma using digital pathology quantitation
Source: BMC Cancer. 2020 Jun 1;20:500. doi: 10.1186/s12885-020-06987-y (PMC7268770; doi:10.1186/s12885-020-06987-y)
Supplement: Supplementary file 2 — Additional file 2: Table S2. R.O.C Cut Offs. [file 12885_2020_6987_MOESM2_ESM.docx]

Supplemental Table 2 – R.O.C Cut Offs

| **Biomarker** | **R.O.C Cut off** |
| --- | --- |
| CD3 | 555.8 |
| CD4 | 440.1 |
| CD8 | 281 |
| CD45RO | 439.6 |
| ICOS | 24.2 |
| PD-1 | Any positivity |
| PD-L1 | Clinical thresholds for NSCLC |
