# Supplementary material for: The adaptive immune and immune checkpoint landscape of neoadjuvant treated esophageal adenocarcinoma using digital pathology quantitation
Source: BMC Cancer. 2020 Jun 1;20:500. doi: 10.1186/s12885-020-06987-y (PMC7268770; doi:10.1186/s12885-020-06987-y)
Supplement: Supplementary file 3 — Additional file 3: Table S3. Discovery Cohort Multivariate Analysis Excluding TRG 1 and 2 cases. [file 12885_2020_6987_MOESM3_ESM.docx]

Supplementary Table S3 – Discovery Cohort Multivariate Analysis Excluding TRG 1 and 2 cases.

| **Multivariate Analysis** | | |
| --- | --- | --- |
| Variables | Discovery | |
|  | HR (95% CI) | p-value |
| Mandard | 0.809 (0.453-1.445) | 0.474 |
| T Stage | 0.734 (0.481-1.119) | 0.150 |
| N Stage | 1.691 (0.833-3.430) | 0.146 |
| Node Pos | 4.304 (1.457-12.712) | **0.008** |
| CRM Involved | 1.583 (0.644-3.893) | 0.317 |
| CD45RO/ICOS | 0.379 (0.179-0.799) | **0.011** |
