# Supplementary material for: The adaptive immune and immune checkpoint landscape of neoadjuvant treated esophageal adenocarcinoma using digital pathology quantitation
Source: BMC Cancer. 2020 Jun 1;20:500. doi: 10.1186/s12885-020-06987-y (PMC7268770; doi:10.1186/s12885-020-06987-y)
Supplement: Supplementary file 4 — Additional file 4: Figure S1. Robust digital pathology workflow. Flow diagram demonstrates conceptually the process of biomarker quantification, strict quality control and data validation steps. [file 12885_2020_6987_MOESM4_ESM.pdf]

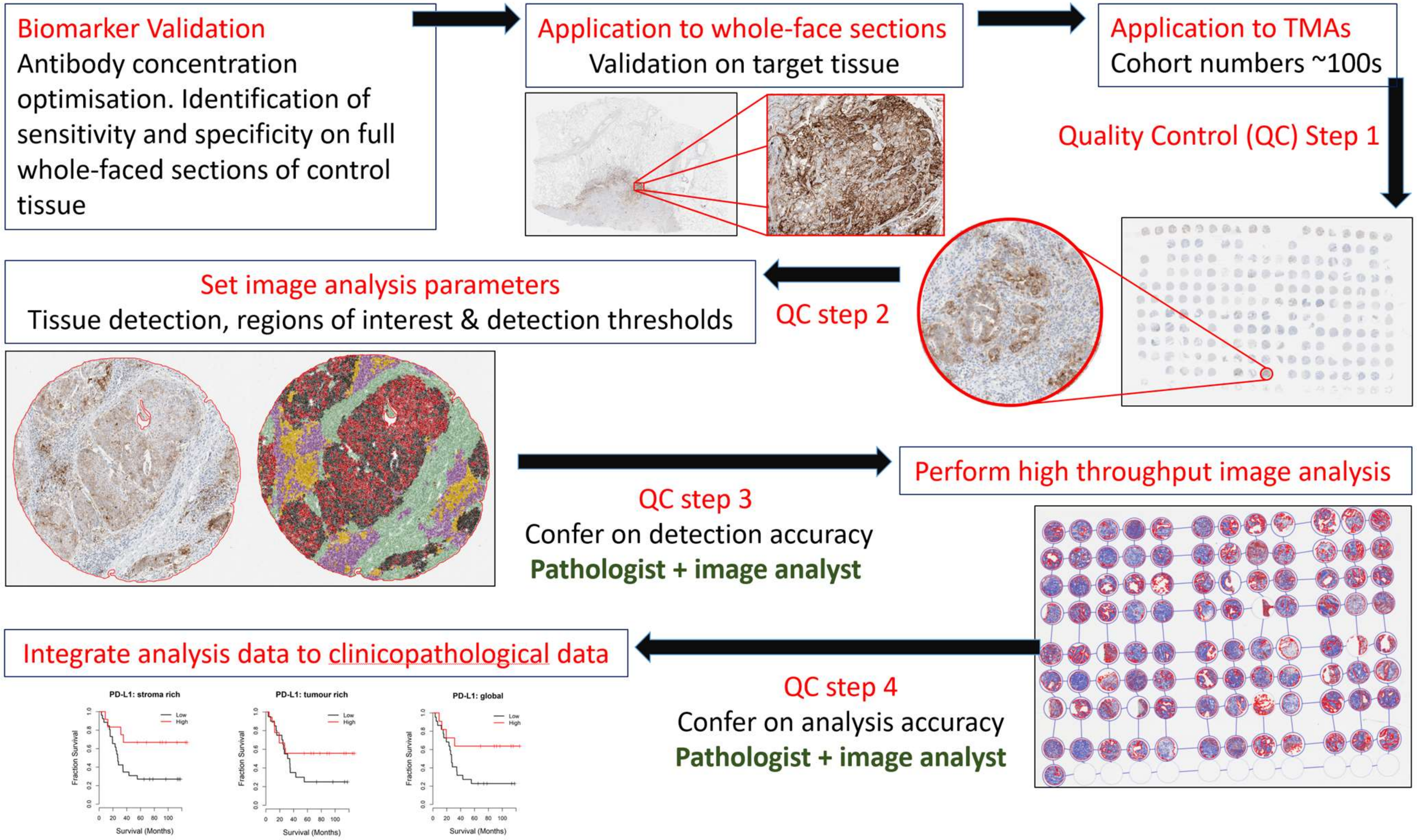

Figure S1. Robust digital pathology workflow. Flow diagram demonstrates conceptually the process of biomarker quantification, strict quality control and data validation steps.
